# Surgical therapy of aorto-iliac aneurysm in a patient with congenital solitary pelvic kidney (CSPK): case report and literature review

**DOI:** 10.1093/jscr/rjad053

**Published:** 2023-02-22

**Authors:** Amedeo Capone, Aaron Thomas Fargion, Davide Esposito, Gianmarco Calugi, Alessandro Alessi Innocenti, Walter Dorigo, Carlo Pratesi, Raffaele Pulli

**Affiliations:** Department of Vascular Surgery, Careggi University Hospital, Florence, FI 50134, Italy; Department of Vascular Surgery, Careggi University Hospital, Florence, FI 50134, Italy; Department of Vascular Surgery, Careggi University Hospital, Florence, FI 50134, Italy; Department of Vascular Surgery, Careggi University Hospital, Florence, FI 50134, Italy; Department of Vascular Surgery, Careggi University Hospital, Florence, FI 50134, Italy; Department of Vascular Surgery, Careggi University Hospital, Florence, FI 50134, Italy; Department of Vascular Surgery, Careggi University Hospital, Florence, FI 50134, Italy; Department of Vascular Surgery, Careggi University Hospital, Florence, FI 50134, Italy

## Abstract

We report a case of a man with an Aorto-Iliac aneurysm and a congenital solitary pelvic kidney (CSPK). The maximum diameter of the aneurysm was 58 mm and the pelvic kidney was perfused by a single renal artery originating from the aortic bifurcation. A computed tomography scan was used for pre-operative planning and the patient underwent aorto-iliac aneurysm replacement with a Dacron graft. The renal artery was reimplanted on the Dacron right limb with a ‘Carrel patch’. Several strategies were adopted to prevent renal ischemia such as sequential aortic cross clamping, selective cold perfusion of renal artery and a temporary Pruitt–Inahara shunt. The post-operative course was characterized by a transient increase in serum creatinine that did not require treatments and the patient was discharged after seven days. Congenital anomalies such as CSPK represent a challenge for the surgeon; however, the adoption of different intraoperative available strategies allowed to reduce possible complications.

## INTRODUCTION

Congenital pelvic kidney is the most uncommon of the six kidney anatomical abnormalities (pelvic, lumbar, abdominal, cephalic, thoracic and crossed) [1]. This anatomical abnormality is more common for the left kidney. Embryologically, a pelvic kidney results from failure of kidney migration between the fourth and eighth weeks of gestation. The incidence of pelvic kidney ranges from 1/2100 to 1/3000 in the general population and its association with abdominal aortic aneurysm (AAA) is extremely rare [2, 3]. This anomaly, so far, is reported in 35 cases of patients who underwent elective surgery for AAA. In addition we present our case report.

## CASE REPORT

**Figure 1:**
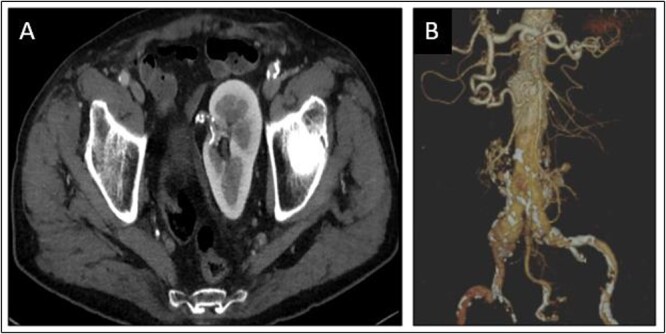
Congenital solitary pelvic kidney (**A**), aortic carrefour and single renal artery (**B**).

An 81-year-old man was incidentally diagnosed with AAA during a routine abdominal echography. His risk factors included active smoking and hypertension. Computed tomography scan confirmed the presence of an aorto-iliac aneurysm and a CSPK (Fig. 1A) supplied from a solitary renal artery arising from the aortic bifurcation (Fig. 1B).

The maximum diameter of the aneurysm was 58 mm, the diameter of the right common iliac artery (CIA) was 29 mm and of the left CIA was 18 mm. Pre-operative blood samples exams were in normal ranges (Hb 13 g/dl, serum creatinine 1.05 mg/dl, urea 0.5 g/l). Cardiological and respiratory pre-operative evaluation for surgical risk assessment were carried out (EF: 55%, FEV1: < 70%) and the patient was judged at mid surgical risk (ASA score grade 3).

**Figure 2:**
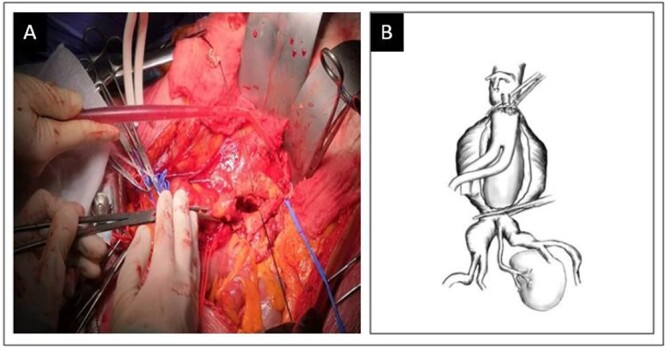
Sequential aortic cross-clamping (**A**) and schematic representation (**B**).

An elective open surgical approach was planned due to the anatomical characteristics of the patient. With a median laparotomy, we proceeded by isolating both the proximal aortic neck, the right and left common iliac arteries, the inferior mesenteric artery and the single renal artery. Before aortic clamping, 30 UI/kg (International-Units) of sodium heparin were intravenously administered. Sequential aortic cross-clamping (Fig. 2) was performed to allow iliac backflow for renal perfusion during proximal anastomosis. Aneurysm was replaced by a Dacron bifurcated graft 16–8–8 mm. After completion of the proximal anastomosis, the distal clamping was moved to the iliac bifurcation on both sides. The aortic aneurysm was opened and a selective cold perfusion (4°C Ringer’s lactate solution) of renal artery was performed after visualization of the vessel ostium. An endarterectomy of the ostium of the renal artery was necessary and it was reimplanted on the right limb of the prosthesis with a ‘Carrel patch’. Cold perfusion was removed and a Pruitt**–**Inahara shunt (positioned between the Dacron right limb and the renal artery) during the anastomosis time allowed a warm perfusion of the kidney. Once the antegrade flow in renal artery was ensured, the distal anastomosis of the graft was completed between the prosthesis limbs and CIAs. In the post-operative course, the patient was admitted to the intensive care unit and a transient rise of serum creatinine (2.2 mg/dl) in the following 5 days occurred. The patient was discharged home in post-operative day 7 without any other complications. At 1-year follow-up, the patency of either the Dacron graft or renal artery was confirmed by Duplex examination; furthermore, renal function continued to be normal as serum creatinine and eGFR were in range.

## DISCUSSION

In literature, several open and endovascular options for patients undergoing surgical treatment for aorto-iliac aneurysm with concomitant pelvic kidney have been reported. In all cases, prevention of renal ischemia was crucial [2].

We included 36 cases in our literature review (Table 1): of these, 6 cases were treated with endovascular surgery, one patient underwent hybrid surgery and 29 were treated with open surgery. Several strategies of renal ischemia prevention have been used; selective cold perfusion and various shunt techniques were adopted in most cases.

**Table 1:** Literature of aorto-iliac aneurysms reported in patients with congenital pelvic kidney

**References**	**No. of patients**	**Side**	**Type of aortic repair**	**Renal protection technique**	**Complications**
Ezzet *et al.*	1	Left	Dacron bifurcated graft	Simple clamping	–
Hans *et al.*	1	Right	Dacron bifurcated graft	Selective cold perfusion	–
Hollis *et al.*	2	Right	Dacron bifurcated graft, 2 of 2 pelvic kidney arteries reimplanted to the main body of the graft	Proximal double clamping during proximal anastomosis and in situ cold perfusion during distal anastomosis	–
		Left	Dacron tube graft, the lower of 2 pelvic kidney arteries included in the distal aortic anastomosis and the upper reimplanted in the left common iliac artery	Selective cold perfusion	
Belcastro *et al.*	1	Right	Dacron tube graft	Distal double clamping	–
Shchneider *et al.*	1	Left	Dacron bifurcated graft, 1 of 1 pelvic kidney artery reimplanted to the right iliac limb of the graft	Proximal double clamping during proximal anastomosis and temporary shunt from the body of the graft to the pelvic kidney artery during distal iliac anastomoses	–
Glock *et al.*	1	Right	Dacron tube graft, 1 of 2 pelvic kidney arteries reimplanted, the other was included in the distal anastomosis	Distal double clamping	–
Kaplan *et al.*	1	Solitary	Endovascular device	–	–
Rehrig *et al.*	1	Right	Dacron bifurcated graft, 1 of 1 pelvic kidney artery reimplanted	Proximal double clamping during proximal anastomosis and temporary shunt from the main body into the pelvic kidney artery during iliac anastomoses	Transient rise in serum creatinine
Faggioli *et al.*	3	NA	Ectopic renal arteries reimplanted or included in the distal anastomosis	Selective cold perfusion if cross-clamp time[40 min, and no protection if the time\40 min	–
Renzulli *et al.*	1	Horseshoe	Dacron tube graft with superior mesenteric artery selective bypass graft	Proximal double clamping during proximal anastomosis and selective cold perfusion to the largest renal artery during distal anastomosis	Transient rise in serum creatinine
Murakami *et al.*	1	Solitary	Dacron tube graft, 2 pelvic kidney arteries included in the distal anastomosis	Selective cold perfusion to the upper pelvic kidney artery	–
Hanif *et al.*	1	Right	Dacron trifurcated graft, selective grafting of 1 of 1 pelvic kidney artery (9-mm graft)	Temporary shunt from the right axillary artery to the pelvic kidney artery during all anastomoses	Transient rise in serum creatinine
Mandolfino *et al.*	1	Right	Dacron bifurcated graft, 2 of 2 pelvic kidney arteries reimplanted to the graft	Simple clamping with systemic administration of dopamine and mannitol	–
Bui *et al.*	2	Left	Dacron bifurcated graft, 1 of 1 pelvic kidney artery reimplanted to the graft.	Simple clamping with cooling	–
		Left	Dacron tube graft	Simple clamping	
Coney *et al.*	1	Left	Dacron bifurcated graft, 2 of 2 pelvic kidney arteries reimplanted to the right iliac limb of the graft.	Temporary shunt from the right axillary artery to the pelvic kidney arteries during proximal and right iliac anastomosis	Thrombosis of one of two arteries supplying pelvic kidney
Marone *et al.*	4	Right	Dacron tube graft, 1 of 1 pelvic kidney artery included distal anastomosis	Selective cold perfusion	One case of atrial fibrillation
		Left	Dacron tube graft, 1 of 1 pelvic kidney artery reimplanted to the graft	Selective cold perfusion	
		Right	Dacron bifurcated graft, 1 of 1 pelvic kidney artery reimplanted to the main body of the graft	Selective cold perfusion	
		Left	Dacron bifurcated graft, 1 of 2 pelvic kidney arteries reimplanted to the main body of the graft, the other was included in the distal anastomosis.	Selective cold perfusion	
Morales *et al.*	1	Left	Endovascular device	–	–
Makris *et al.*	1	Solitary	Dacron tube graft, 1 of 2 pelvic kidney arteries included in the distal anastomosis	Temporary shunt from the right axillary artery to the right femoral artery and a second shunt from the right common iliac artery to the pelvic kidney artery during all anastomosis	Pneumonia
Spear *et al.*	1	Left	Endovascular device	–	Transient rise in serum creatinine
Jinnouchi *et al.*	1	Right	Dacron trifurcated graft, selective grafting of 1 of 1 pelvic kidney artery (8 mm graft)	Selective cold perfusion	–
Akashi *et al.*	1	Right	Dacron tube graft, 1 of 2 pelvic kidney arteries reimplanted to the graft	Selective cold perfusion	–
Date *et al.*	1	Right	Dacron bifurcated graft, 3 of 3 pelvic kidney arteries reimplanted to the right iliac limb of the graft	Selective cold perfusion	–
Malinowski *et al.*	1	Solitary	Dacron bifurcated graft, 2 of 2 pelvic kidney arteries reimplanted to the right external iliac artery + EVAR	Selective cold perfusion	Type II endoleak
Saito *et al.*	1	Solitary	Dacron bifurcated graft, 1 of 1 pelvic kidney artery reimplanted to the right limb of the graft	Brewster’s method and Selective cold perfusion	–
Majumder *et al.*	1	Right	Fenestrated Endograft	–	–
Alves Ramos Diniz PI *et al.*	1	Left	Dacron trifurcated graft, selective grafting, 1 of 1 pelvic kidney artery	–	–
Ertugay *et al.*	1	Solitary	Evar chimney	–	Type II endoleak
Centofanti *et al.*	1	Left	Evar branched	–	
Present case (2021)	1	Solitary	Dacron bifurcated graft, 1 of 1 pelvic kidney artery reimplanted to the right limb of the graft	Double proximal clamping and selective cold perfusion during proximal anastomosis and temporary shunt during renal replanting	Transient rise in serum creatinine

The endovascular options do not require the prevention of renal ischemia but, in most cases, involve advanced techniques.

Morales *et al.* reported a type IV thoraco-abdominal aortic aneurysm with congenital pelvic kidney. In this case treatment required deployment of a custom-made endoprosthesis with a fenestration for superior mesenteric artery, right renal artery and at the aortic bifurcation where the artery that supplied pelvic kidney originated [1].

Majumder *et al.* also reported a case of abdominal aortic aneurysm treated with a fenestrated custom-made endoprosthesis [4].

In two other cases endovascular surgery was chosen to treat AAA with CSPK. In one case the patency of the renal artery was ensured by the chimney technique and in another by Iliac Branch Device [9, 10].

Double proximal or distal clamping take advantage of collateral circles (lumbar artery, inferior mesenteric artery and iliac arteries backflow). This technique has been used in a minority of cases [5, 6].

A second option reported is the placement of a temporary shunt [5–9]. This is an invasive procedure that can be complicated by either bleeding, thrombosis and dissection.

The placement of a temporary shunt was also subject to variability within different cases reported in the literature. In most cases, an axillo-renal shunt was preferred (Brewster’s method), whereas, in others, it was placed between the prosthesis and the renal artery after the proximal anastomosis was made.

The most common strategy was selective cold perfusion of renal artery [2, 7]. In a series of three cases, cold perfusion of renal artery with Ringer’s lactate solution was used only in case of clamping longer than 40 min. In the present case, prevention of renal ischemia was carried out with different strategies during surgery: first a sequential aortic cross clamping technique, secondly a selective cold perfusion and then a Pruitt**–**Inahara shunt between the left leg of the prosthesis and the renal artery to complete graft implant.

In all cases, a follow-up by Duplex ultrasound or CT-scan was performed. The patency of the renal arteries supplying the pelvic kidney was guaranteed in most patients. Only one case of renal artery thrombosis was reported in a patient who fortunately had two renal arteries [6]. In four other cases, in addition to ours, a transient rise in serum creatinine was detective in the post-operative period [5]. One patient had a heart complication (atrial fibrillation) and another one had pneumonia, which required intravenous antibiotic therapy [3]. Type II endoleak was reported in two patients undergoing endovascular treatment and did not require reintervention [7].

In concomitant aorto-iliac aneurysm and CSPK, pre-operative planning is crucial. The gold standard technique does not exist and different ‘surgical tips and tricks’ should be considered. Each single case should be dealt with taking into account both the operator experience and its ability to combine different techniques.

## CONFLICT OF INTEREST STATEMENT

The authors certify that there is no conflict of interest with any financial organization regarding the material discussed in the manuscript.

## FUNDING

None.
